# Therapeutic impact of determination of RAS mutations in the plasma of patient with colorectal cancer 

**Published:** 2022

**Authors:** F Macedo, J Monteiro, T. Cunha Pereira, A.R. Monteiro, R. Felix Soares, N Bonito, G Sousa

**Affiliations:** *Medical Oncology Department, Portuguese Oncology Institute of Coimbra, Portugal*

**Keywords:** colorectal cancer, RAS gene, real-time PCR, circulating tumor cell, molecular targeted therapies

## Abstract

Stage IV colorectal cancer treatment includes targeted therapy depending on RAS status. During disease progression, loss or gain of RAS mutations could happen, supporting the hypothesis of the evolutionary pressure of therapy. Circulating tumor DNA (ctDNA) are nucleic acids released to the bloodstream by the tumor during its development and may be detected by liquid biopsy.

The Idylla© Biocartis, a fully automated real-time-PCR-based molecular diagnostic system, was used in a patient with metastatic colorectal cancer with a NRAS mutation in progression after several therapeutic lines. The ctDNA mutational analysis was performed and revealed the absence of mutations in the KRAS, NRAS, and BRAF genes. The patient started the third line of palliative chemotherapy with irinotecan + cetuximab and achieved a partial response for the first time. The authors describe a case in which liquid biopsy determined the higher progression-free survival achieved.

## Introduction

 Colorectal cancer (CRC) is the third most commonly diagnosed malignancy and the fourth leading cause of cancer death in the world, accounting for about 1 million new cases and almost 550,000 deaths worldwide in 2018 (1). It is expected that the global burden of CRC will increase by 60% in 2030 (1).

For every case of CRC diagnosed, about 20% is metastatic at diagnosis, and some of the cancers treated with curative intent will became a stage IV disease some years later (2). The treatment for stage IV disease depends on whether we are facing an oligometastatic disease, which could be offered some locoregional treatments, or a metastatic disease, for which the only option is a systemic treatment. The systemic treatment for metastatic CRC (mCRC) includes the conventional chemotherapy (based in fluoropyrimidines, irinotecan, and oxaliplatin), and targeted therapy (based in epidermal growth factor receptor inhibitors (EGFR) and vascular endothelial growth factor inhibitors (VEGF)). This kind of combination increases the overall survival rates and progression free survival (3).

Cetuximab and panitumumab are two EGFR-targeted monoclonal antibodies, and cetuximab is approved as the first-line therapy for RAS wild-type mCRC (4). The RAS family is comprised of proteins linked to proliferation and invasion and includes KRAS, NRAS, and HRAS. When one of these proteins is mutated, the receptor becomes constitutively activated, leading to more aggressive CRC. RAS mutation predicts no response to EGFR inhibitors, so afflicted patients will not benefit from that kind of treatment. KRAS mutation is present in mCRC in about 40% of cases, NRAS in about 3%, and HRAS is very rare (5).

It was found that during treatment with cetuximab, approximately 50% of tumors that are initially RAS wild type will acquire resistance to this compound, and the disease will progress. Accordingly, in tumors progressing during cetuximab treatment, it will become important to determine if there are new mutations (6).

During its development, cancer releases some nucleic acids to the bloodstream called circulating tumor DNA (ctDNA), which contain all the genetic alterations that the tumor is suffering over time. A new technique has been developed to detect this material in blood samples, i.e. liquid biopsy (7). Some studies have already proven the concordance between the results obtained from liquid biopsy and those obtained from tissue (8). One advantage of this method is its minimally invasive technique, which makes it acceptable to perform continuous monitoring of the tumor behavior and avoid restriction to tissue-based mutations. A disadvantage of it is the fact that ctDNA is only a small portion of all existent DNA (9). 

## Case Report

**Figure 1:**
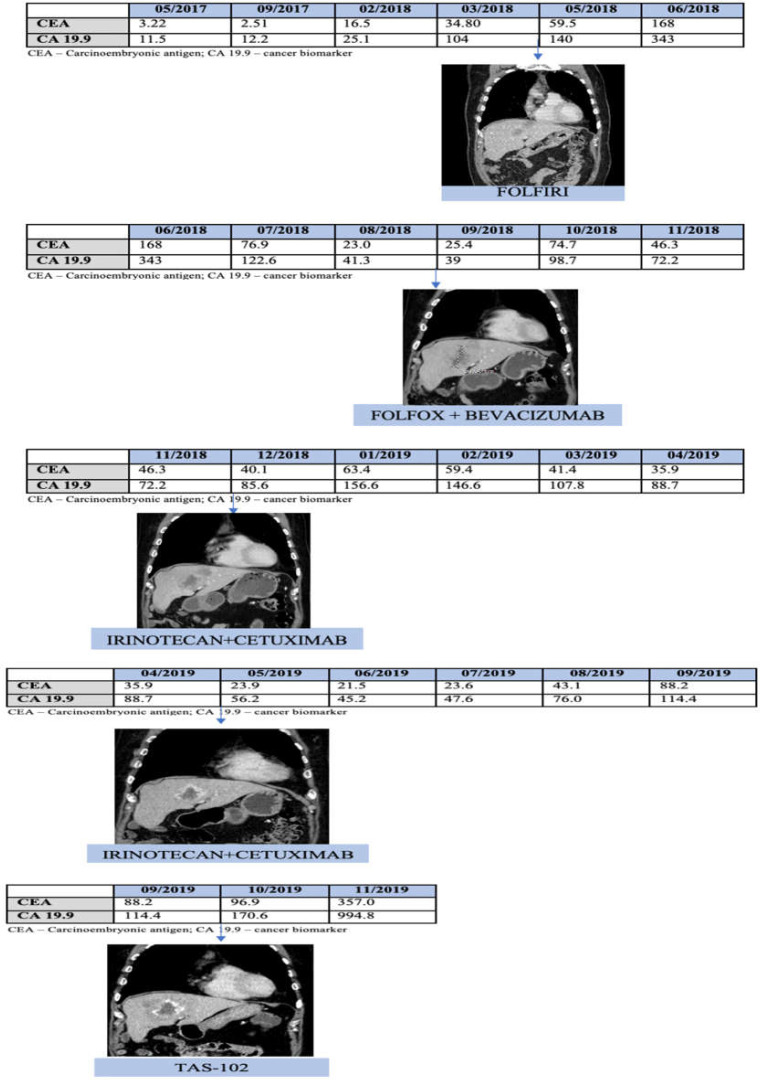
Tumoral biomarkers and computed tomography evolution

Herein, the authors present the case of a 75-year-old male with a medical history of depression, arterial hypertension, prostate cancer treated with radiotherapy only in 2003 resulting in a radic cystitis and nefrectomy due to renal tumor in 2006 (creatinine clearance of 63 mL/min). The patient was medicated with carvedilol, nifedipine, metildopa, and chlortalidone. In July 2016, the patient presented with hematochezia and no pain. As this symptom was maintained in time, a colonoscopy was performed and revealed a tumor with 25 mm extension, 65 cm from the anal margin. The clinical staging was cT2N0M0. The patient had a right hemicolectomy in September 2016 with the following histology: invasive adenocarcinoma low grade (G2), with linfovascular invasion and metastasis in one lymph node from 25 (1/25) – pT3N1a, stage IIIB. From November 2016 until May 2017, the patient completed 8 cycles of adjuvant chemotherapy with capecitabine 1000 mg/m2 (a total of 1800 mg twice a day, 14 days), and he remained under surveillance. The evolution of the tumoral biomarkers is listed in Figure 1. 

Because of elevation in tumoral biomarkers, an urgent computed tomography (CT) was performed and showed hepatic metastization potentially resectable on IV segment with 31 x 28 mm. The biopsy of this lesion confirmed colorectal metastasis with NRAS mutation in codon Q61, BRAF wild-type, MSI low. In April 2018, palliative chemotherapy was started with folfiri in monotherapy, as the patient had contraindications for bevacizumab (acute renal failure and microalbuminuria). The analytic response was evident with the decrease of CEA from 168 to 76.9 and CA from 343 to 122.6. At the end of 8 cycles of folfiri, a control CT scan was performed to evaluate the response. The results showed progression of the disease with dimensional increase in the lesion (to 49 x 36 mm by RECIST 1.1 criteria). The patient started a second-line palliative chemotherapy with FOLFOX + bevacizumab, because the renal function of the patient was now normal, and he had no microalbuminuria. The patient completed 5 cycles of FOLFOX + bevacizumab with good tolerance and was diagnosed with diabetes mellitus. In December 2018, a new control CT scan was performed and showed disease progression (increase of hepatic metastatic lesion, now with 58 x 48 mm, by RECIST 1.1 criteria).

## Methods

The Idylla© Biocartis was used, a fully automated real-time-PCR-based molecular diagnostic system. About 1 mL of plasma was used for the analysis of Kras and Nras mutations. The ctKRAS mutation assay allows the detection of 21 mutations, and the ctNRAS/BRAF mutation assay permits the detection of 18 mutations in the NRAS gene and 5 mutations in the BRAF gene. The hands-on time was less than 2 minutes, and automatic reporting took 130 minutes. A sample is considered as positive for mutation when the PCR curve is under the validated range. If the sample is not within this range, the patient is considered wild type (no mutation detected). 

## Results

In December 2018, ctDNA mutational analysis revealed the absence of mutations in KRAS, NRAS, and BRAF genes. In January 2019, the patient started the third line of palliative chemotherapy with irinotecan + cetuximab. He completed 6 cycles of treatment, and the subsequent control CT scan showed partial response (dimensional decrease to 45x35 mm with peri-lesional necrosis, by RECIST 1.1 criteria). It was the first time the tumor had responded to palliative chemotherapy. The patient presented a progression free survival with irinotecan + cetuximab of 9 months, which was the maximum achieved with all the treatments made. In September 2019, a new progression was documented by CT scan, and a fourth line was initiated with trifluridine + tipiracil.

**Figure 2:**
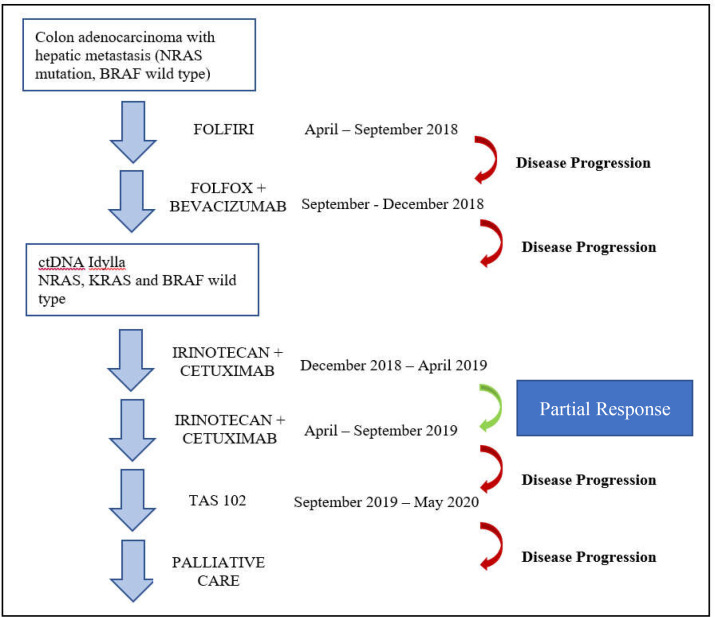
Clinical and therapeutic history of the patient

## Discussion

RAS mutation is a powerful negative predictive biomarker for the response to anti-EGFR therapy. In RAS mutant CRC, first-line treatment is a chemotherapy doublet (FOLIFIRI, CAPOX, FOLFOX) in combination with the monoclonal antibody bevacizumab, which is used until progression or toxicity. Once these doublets are used, other lines available are aflibercept, TAS-102, regorafenib, or ramucirumab (3).

It was shown that the analysis of ctDNA in blood samples had a high concordance with the standard tissue biopsy, giving the possibility to monitor tumoral heterogeneity during treatment in a non-invasive way (10). The tumor RAS mutations status can switch between mutated and wild type because of the evolutionary pressure of treatments by the positive or negative selection of clones (11). The patient described herein was a primary RAS mutant mCRC who was treated accordingly, and his RAS status had changed to wild-type due to the selection of clones after first-line treatment failure. When he received a third line with anti-EGFR therapy, the patient achieved the most durable clinical benefit compared with all other therapies made (Figure 2). 

Some questions should be raised: 1) As there are already some cases in the literature that switched their RAS status and benefited from anti-EGFR therapy (12), should the molecular identity of the tumor be checked at each progression? 2) The limit value for detection of RAS mutations in plasma is about 1-5%, so a negative test cannot exclude that these clones are present in a small portion and the patient will benefit from anti-EGFR therapy; 3) A negative test could also be due to an insufficient amount of ctDNA in the blood sample. 

Several studies have tried to clarify the role of clearance of RAS mutated clones under anticancer treatment. In the PLACOL study, 61 patients with primary RAS mutated tumor were included and RAS status in plasma was monitored by NGS or PCR in first progression. In 22.2% of patients with RAS mutation detected in plasma at inclusion, no mutation was detected after disease progression. However, only 0.03% had positive methylated markers, which suggests that the clearance of RAS mutated clones under anticancer treatment is a rare event (13). Raimondi et al. described a small group of 11 patients with RAS mutated cancer, of which 45% switched to RAS wild type during treatment, but only one had proven clearance (14). In their study, however, the mutational status of ctDNA was not assessed before treatment was initiated. It is important to reflect that the loss of RAS mutated clones in plasma is not synonymous with a real clearance in tissue. It is, therefore, crucial to assess the presence of ctDNA. Another similar study was conducted by Klein-Scory et al., in which 12 patients with mCRC classified as RAS mutated and BRAF wild type by NGS were included. Notably, all patients with partial response or stable disease had conversion to RAS wild type. Mutational frequency decreased after 4-5 cycles of therapy (15).

Sunakawa et al. demonstrated the conversion of RAS mutated to wild type in plasma in 76% of patients (16). Spindler et al. reported a conversion rate of about 27% from RAS mutated to wild type in the moment of disease progression after second line therapy (17). Vidal et al. showed that patients with baseline RAS mutations had decreased mutation load after 8-12 weeks of treatment (18). Li et al. found significantly less RAS mutation in samples after chemotherapy (43.8%) when compared to samples without chemotherapy (54.5%) (*p* = 0.043) (19).

Bouchahda et al. concluded that patients with RAS mutated mCRC whose plasma biopsies contained RAS wild type clones could benefit from cetuximab-based therapy. They demonstrated that 56% of patients who had RAS wild type in the ctDNA were RAS mutated in solid tumor tissue before, supporting the theory of possible loss of such RAS mutation over time in heavily pretreated patients (20).

There has been some concern regarding the discrimination between patients with real clearance of RAS mutation in plasma from those maintaining some mutated clones. For this purpose, a colon cancer specific gene methylation panel has been tested. The methylation test confirmed the presence of ctDNA in most RAS wild-type samples at the time of disease progression, thus confirming that the negative selection of RAS mutant clones during the clonal evolution of mutant RAS colorectal cancer is not an infrequent event (21).

The KAIROS trial aimed to determine whether the response to EGFR inhibition in patients with RAS mutant cancers converted to RAS wild type during the course of treatments might become the rule rather than the exception. Unfortunately, the KAIROS trial was closed. The authors hope that the planned MoLiMor trial will help answer some of these questions. In this phase II trial, patients with RAS mutant mCRC who converted to RAS wild type will be submitted to the intermittent addition of cetuximab, and RAS mutation status will be monitored by liquid biopsy.

## Conflict of interests

The authors declare that they have no conflict of interest.
